# China-Pakistani economic corridor project bring the international trade, healthcare, self-efficacy, and social performance facility to Gilgit city, Pakistan

**DOI:** 10.1016/j.heliyon.2022.e10523

**Published:** 2022-09-03

**Authors:** Shakela Naz, Wu Yeyan, Liu Zhe, Keyao Ren, Yu Wenjie

**Affiliations:** a College of History, Nanjing University, 163 Avenue, Xianlin Comps, Nanjing, 210093, China; b Nanjing University Collaborative Innovation Center of South China Sea Studies, 22 Hankou Street, Gulou, Nanjing, 210093, China; c School of Marxism, Changshu Institute of Technology, Changshu, China

**Keywords:** Gilgit city, History of CPSR, Healthcare, International trade, Social performance

## Abstract

This paper aims to identify the impact of China-Pakistani economic corridor project bring the international trade, healthcare, self-efficacy, and social performance facility to Gilgit city, Pakistan. A questionnaire survey was carried out on the scholar presently studying at different universities in china. The 50% scholar estimation silk road enhanced, and 11% have no idea about the increasing of the healthcare opportunity in Gilgit. The 83% scholar estimation silk road improved Gilgit infrastructure. The 66% scholar estimated yes, 10% no, and 50% have no idea about the silk road brings peace and stability to Gilgit. The majority of respondents in the survey had a positive approach to the China–Pakistan Silk Road (CPSR). The results of the analysis show a better knowledge of the communication between the project and social media. This study will help china intensify its products to the middle east, africa, and europe. The (CPSR) is believed to create a shift in china's economic and political position in the world. in addition to strengthening the time-tested economic partnership between the two countries, the CPSR is expected to transform china's economic and political situation in the world.

## 1 Introduction

China-Pakistan friendships begin in 1950 when Pakistan was in the midst of the first country to end representative political relationships with the People's Republic of China. Since then, both countries have positioned significant importance on maintaining too close and supportive friendships (Masood, 2008). Bilateral associations contain evolved from a primary Chinese policy of impartiality to a corporation with a minor but militarily powerful in Pakistan. Diplomatic relationships were recognized in 1950; military assistance begin in 1966, and an intentional agreement was signed in the mid of 1972. The economic collaboration between them starts in 1979. China has the biggest arms supplier to Pakistan and its third-largest trading collaborator (Li and Hans-Jörg, 2017; Ejdys et al., 2017).

In addition, the investment will be heavily focused on the development of the China-Pakistan Economic Corridor (CPEC), transport and energy projects, and a deep-sea port that offers direct access to the Indian Ocean and beyond. Even though the two parties expect a common advantage from the scheme, many experts fear that project's completion will benefit China more than Pakistan. Pakistan's limited prospects benefit from challenges around the project. Political instability, poor administrative presentation, local ambiguity and geopolitical illustration, and grave security problems are probable to challenge the public interest (David et al., 2016; Alhassan et al., 2021). Good health care and quality of life are said to be a global asset and interconnected, meaning that health and economic growth are intertwined, making it a key element of sustainable economic growth (Alhassan et al., 2021). The country's economy is driven by building a healthy population, to understand the income of future generations; the life cycle model explains the relationship between the health status of citizens, wealth and expenditure (Hossain et al., 2021). Good healthcare services that reduce mortality at all levels and lead to partnerships for sustainable economic growth, economic growth, sustainability, education, and goal attainment (Alhassan et al., 2021; Hossain et al., 2021).

By analyzing existing publications and public media outlets, the present study aims to recognize the significant challenge and build up a roadmap that how Pakistan can take enhanced advantage of the performance of the Karakoram Highway (KKH) Silk Road. Economist Dr Qaiser Bengali says the move to dismantle militant hideouts in the northwest, where Pakistan faces many challenges, has created an atmosphere of hope, and at the moment. The arrival of Chinese investment shows that Pakistan has once again had to compete the opportunity for economic change in life. Experts state it will produce jobs and financial movement in Pakistan, which has been a failed system of military power and service delivery for the past three decades, torn apart by armed insurgency (Hossain et al., 2021). The objectives of the present research (1) To know about the significance of the PCSR project. (2) To check the balance between the two nations, Pakistan and China. (3) To know about the most critical risk and challenges associated with this project (Alhassan et al., 2021). The present study aims to identify the impact of China Pakistan Silk Road's performance on international trade. It also expects to develop an infrastructure for how Pakistan can make sure its economic benefits. As to the Silk Road's significance, most of the respondents considered that the planned projects' operationalization would create jobs, provide infrastructure, and encourage domestic and international investment flow to Pakistan. We have investigated three essential issues: “1st the projects' significance. 2nd is a balance in advantages between the two countries, and 3rd is the majority of critical risks and challenges connected with the scheme. Or The present study aims to identify the impact of China-Pakistani economic corridor project bring the international trade, healthcare, self-efficacy, and social performance facility to Gilgit city, Pakistan.

## 2 Methodology

### 2.1 Ethics approval

This paper is extracted from PhD thesis and approved by advanced board of research studies and departmental research committee of the College of History, Nanjing University, China. This study is not harmful for animals and humans in any manners. The collected data for this study is solely used for research purpose.

### 2.2 Data collection

This study was quantitative and questionnaire was used for data collection. The questionnaire has been filled up and informed to all participants about research topics that it has been used for research purposes. A questionnaire was spread by Facebook, QQ international, Wechat, WhatsApp, by face to face interview, etc (Appendix A, Appendix B, and Appendix C) the information has been acquired from significant sources, including the State Bank of Pakistan's Economy, Chinese economic databases, and the urban numerical annual report. The Chinese Urban numerical annual report is a popular numerical annual report published annually by the Chinese National government department of information.

**Figure 1:**
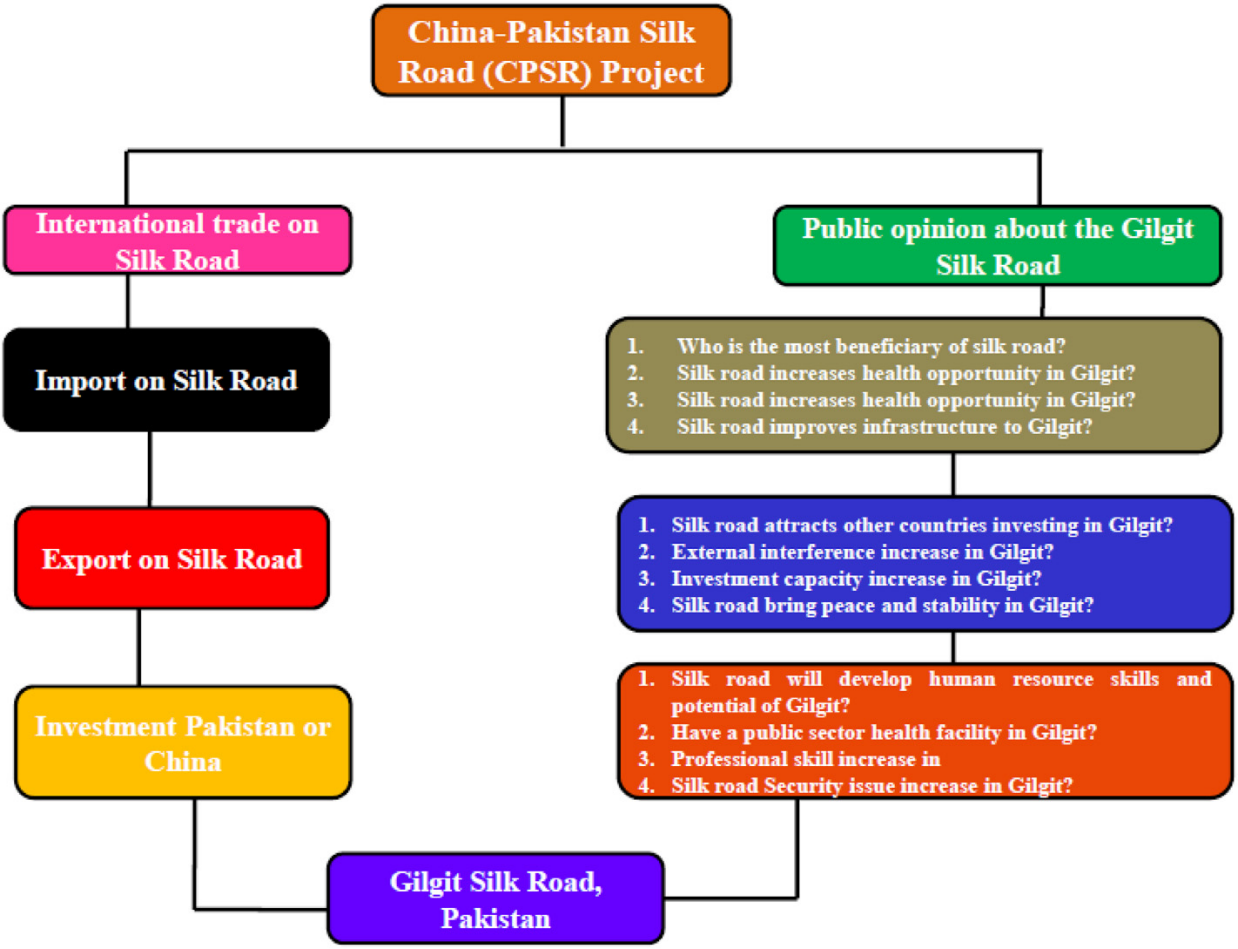
The study model justification.

A purposive sampling technique was used to select the target population for this study. The main purpose of the purposive sampling is to collect the opinion of Pakistani students studying in China was used to identify China-Pakistan Silk Road's significance and challenges. Because they have more knowledge on this topic than those students studying in Pakistan. The students' direct and indirect involvement and thoughtful of the Chinese civilization them a dependable source of information. Sentiments of 648 students were collected on three essential issues (Table 4): “1st the projects' significance. 2nd is a balance in advantages between the two countries, and 3rd the majority of critical risks and challenges connected with the scheme (Figure 1).

### 2.3 Statistical analysis

The collected data were analyzed with the help of SPSS 22nd version randomized complete block design (RCBD) and the excel information and communications technology (ICT) analysis. We have used the scale of percentage (%) as statistically significant.

## 3 Results and discussion

### 3.1 International trade in China-Pakistan silk road

China's new development model is no longer based on industrial production, imports and exports, cheap labor, and other than the improvement of domestic expenditure and services, this paper is analysis the financial system of the countries not just as shoppers of the Chinese product but also a commerce partners and supplier of products on the Chinese market (Khan et al., 2013). For this purpose, it had been stranded on normative theory important the information of the countries' evolution and allowing for the role of exports in the GDP (Khalil and Perveen, 2015; Oziewicz, 2015). Such a point of view looks to be necessary also as a result of one of all the most considerable challenges within the economic relationships of Asia and European countries with China is their quickly increasing trade income with the member states and considerably higher development of imports compared to exports to China (Li, and Hans-Jörg, 2017). In the present study, nine (9) foreign direct investments (FDI) are illustrated in Figure 1a. Under the CPEC, FDI has been slowing down after steady growth over the past three years. The inflows reduced by 19.2% in H1-FY19 over the last year's corresponding period near the end of the early harvesting projects. Investment in the CPEC-related power sector is now shifting to the transmission and distribution side. By source, China dominated direct investment with a 57.6% stake in net FDI during H1-FY19. In addition to electricity and construction, Chinese investment in the electrical machinery and business sectors was also involved. After China, direct investment from the UK increased to 116.0 million US dollars in H1-FY19, especially in the food and business system (Table 1).

**Table 1:** Net foreign direct investment inflows to Pakistan.

US Dollar in Million	FY18			FY19			Change in FY19	
	Q1	Q2	H1	Q1	Q2	H1	Q2	H1
Oil & gas	52.8	53.9	106.7	74.1	60.6	134.7	6.7	28.0
Power	205.3	406.6	611.9	92.4	109.5	201.9	−297.1	−410.0
Construction	124.8	226.1	350.9	180.3	107.5	287.8	−118.6	−63.1
Telecommunications	62.8	−78.0	−15.3	−54.2	−80.9	−135.1	−2.9	−119.9
Business system	190.1	86.0	276.1	39.8	163.5	203.4	77.5	−72.8
Electrical machinery	0.8	10.2	10.9	5.2	119.5	124.7	109.3	113.8
Others	128.7	162.2	290.9	221.2	280.7	501.9	118.5	211.0
**Total FDI (net)**	**765.2**	**867.0**	**1,632.2**	**558.9**	**760.3**	**1,319.2**	−**106.6**	−**313.0**

Data source: Pakistan Bureau of Statistics. FY: Full-year; Q: quarter; H: half-year

**Figure 2:**
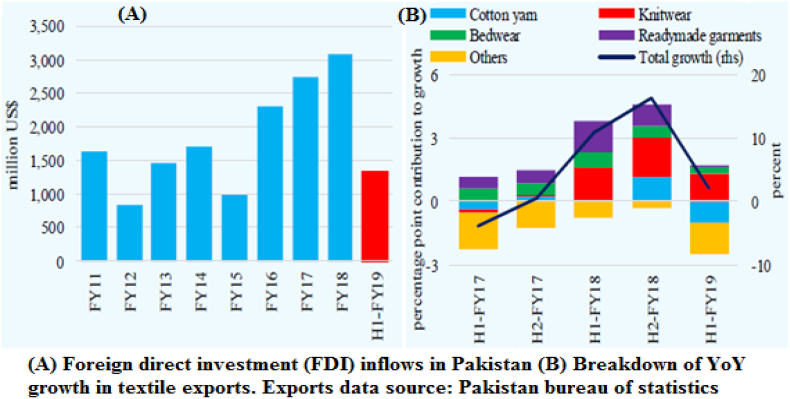
(A) Foreign direct investment (FDI) inflows in Pakistan, (B) Breakdown of YoY growth in textile exports. Export data source: Pakistan Bureau of Statistics.

Pakistan's exports to H1-FY19 increased 1.9 percent to US dollar 11.2 billion after a 10.9% YoY increase in the equivalent period last year. The main reason for this was the rapid rise in the textile sector's exports. The second quarter also showed a significant image, as textile exports refused for the first point in the first quarter. Export lower unit prices met a healthy increase in their quantum exports to a large extent by suppressing the export prices of essential knitwear and readymade garment parts (Table 2 and Figure 2A and B).

**Table 2:** Pakistan's Major Exports during H1.

	Abs. change					Contribution to Growth	
	FY18	FY19	FY19	Quantum impact million US$	Price impact percentage points	FY18	FY19
**Food group**	**1,935.1**	**1,994.9**	**59.8**	**-**	**-**	**2.8**	**0.5**
Basmati rice	203.7	244.2	40.5	53.0	−12.5	0.3	0.4
Non-basmati	645.9	573.8	−72.1	−101.6	29.5	1.1	−0.7
Wheat	0.0	53.5	53.5	126.4	−72.9	0.0	0.5
Sugar	181.5	79.5	−102.0	−85.4	−16.7	0.0	−0.8
Seafood	200.6	183.6	−17.0	−9.7	−7.2	0.17	−0.2
Fruits & veg.	243.9	312.0	68.1	109.0	−41.0	−0.13	0.6
**Textile group**	**6,641.6**	**6,644.3**	**3.1**	**-**	**-**	**5.0**	**0.0**
Raw cotton	53.3	14.1	−39.2	−38.9	−0.3	0.2	−0.4
Cotton yarn	661.5	548.4	−113.1	−128.9	15.8	0.04	−1.0
Cotton fab.	1,066.9	1,052.3	−14.6	287.9	−302.4	0.0	−0.1
Knitwear	1,334.6	1,475.6	141.0	165.2	−24.1	1.6	1.3
Bedwear	1,124.4	1,161.2	36.8	182.3	−145.5	0.7	0.3
Towels	385.8	378.0	−7.8	−51.9	44.0	0.1	−0.1
R. garments	1,249.4	1,259.7	10.3	328.0	−317.7	1.5	0.1
**Other Manuf.**	**1,702.3**	**1,708.1**	**5.8**	**-**	**-**	**1.8**	**0.2**
Leather	159.7	128.3	−31.4	−28.1	−3.4	−0.1	−0.3
Leather manuf.	265.2	247.4	−17.8	−5.7	−12.2	0.1	−0.2
Plastic	114.3	155.7	41.4	2.0	31.0	0.2	0.3
Cement	104.8	157.0	52.2	65.8	−27.4	−0.27	0.4
**POL group**	**163.6**	**269.5**	**106.0**	**-**	**-**	**0.8**	**1.0**
Crude oil	75.2	145.9	70.7	37.4	33.3	0.4	0.6
**All other items**	**533.8**	**564.4**	**30.6**	**-**	**-**	**-**	**-**
**Total exports**	**10,976.4**	**11,181.2**	**204.8**	**954.4**	−**817.0**	**10.9**	**1.9**

Data source: Pakistan Bureau of Statistics. FY: Full-year; Q: quarter; H: half-year

**Figure 3:**
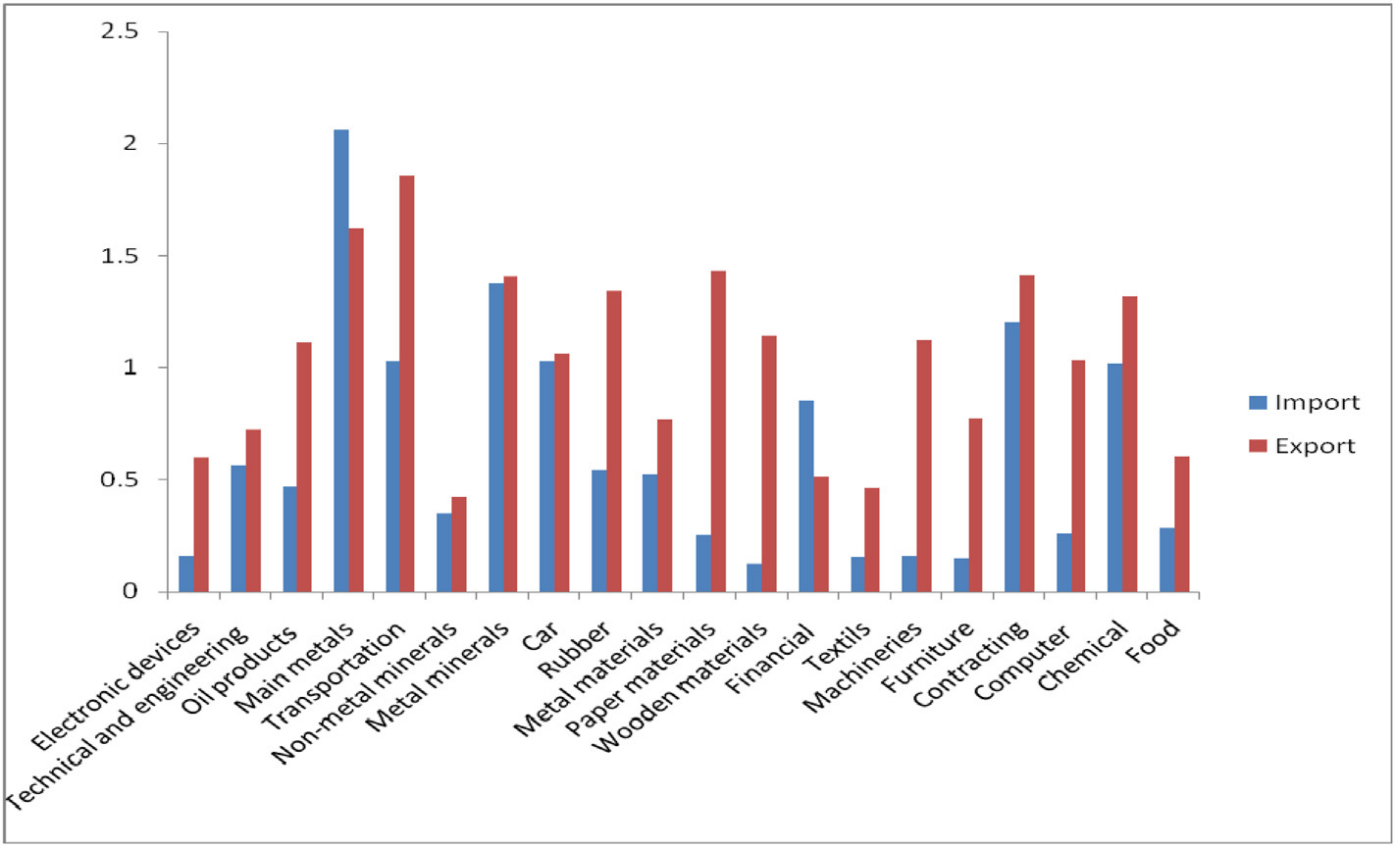
The beta variable's numerical data was used for the 20 industries import and export collected by monthly data as 84 observations.

On the contrary, yarn and cotton decreased due to a heavy quantum fall in their export values. They were also responsible for exporting critical agricultural commodities, namely sugar, wheat, and rice, in small quantities. In sugar and wheat, exporters could not compete in the international market after subsidy subsidies (Figure 3).

In H1-FY19, Pakistan's imports declined for the first time in half a year since H1-FY16, as international stabilization mechanisms are put in place and the economy shows signs of slowing down. The sharp decline in non-energy imports represents an increase in the price of energy imports. Imports of power, electrical, and production equipment, besides railway locomotives, were noticeably minor this year in the come around of a significant drop in PSDP payments and conclusion of before time CPEC scheme (Table 3).

**Table 3:** Pakistan's Major Imports during H1 (million US dollars).

Items	FY18	FY19	Abs. change	Quantum impact	Price impact
**Energy group**	**6,675.1**	**7,665.0**	**989.9**	−1,335.8	2,325.6
POL prods.	3,881.2	3,415.1	−466.1	−1,384.8	918.8
Crude	1,756.1	2,426.0	669.9	−170.7	840.6
LNG	876.2	1,709.3	833.1	271.3.1	561.8
**Agri and chem**	**4,278.8**	**4,584.1**	**305.3**	-	-
Fertilizer	512.8	646.2	133.3	53.0	80.3
Plastic mat.	1,090.4	1,080.4	−10.0	26.7.1	−36.8
**Transport group**	**2,012.6**	**1,507.6**	**-505.0**	-	-
Cars	670.4	582.8	−87.6	-	-
Truck & buses	305.1	282.7	−22.4	-	-
Aircraft & ships	358.0	166.3	−191.7	-	-
**Metals group**	**2,566.1**	**2,500.4**	−**65.7**	-	-
Steel scrap	777.3	765.6	−11.7	−41.7	30.1
Iron & steel	1,135.9	1,124.6	−11.3	−30.6	19.2
**Food group**	**3,242.1**	**2,966.2**	−**275.9**	-	-
Tea	283.6	301.7	18.1	70.1	−52.0
Palm oil	1,037.4	931.7	−105.7	90.9	−196.7
Pulses	269.9	287.8	17.9	146.8	−128.9
**Textile group**	**1,378.5**	**1,344.5**	−**34.0**	-	-
Raw cotton	97.1	120.2	23.1	12.3	10.8
Syn. yarn	321.5	302.2	−19.3	−26.9	7.7
**Machinery group**	**5,501.9**	**4,479.3**	−**1,022.6**	-	-
Power gen	1,239.3	661.9	−577.4	-	-
Electrical	1,075.1	881.2	−198.9	-	-
Construction	190.2	129.8	−60.4	-	-
Cell phones	376.5	364.0	−12.4	-	-
Other machinery	1,678.3	1,606.6	−71.7	-	-
**All other items**	**2,386.9**	**2,394.2**	**7.3**	-	-
**Total imports**	**28,694.9**	**27,952.5**	−**742.4**	−**1,144.4**	**2,015.5**

Data source: Pakistan Bureau of Statistics. FY: Full-year; Q: quarter; H: half-year

The hold-up in production action shortened the order for import of raw resources by the household steel industry. The lower transport import contributes to a sizable drop in transport import, accentuating the impact of a go down in pay for railway and aircraft correlated pieces. In addition, lower global palm oil prices covered import values, even though enhanced in the commodity's quantum import throughout the episode. The multinational and quantum-led reduced speed of import growth implies that in Pakistani rupees (Rs.) 24.1% reduction since Nov 2017 and other required density dealings have impacted. The growth in imports of customer substance and related raw substance chop to presently 4.0% YoY in this year, besides a large amount 12.9% increase documentation the previous year. (Khan et al., 2013) similar results reported that the import and export of the electronic devices, technical and engineering, oil products, primary metals, and transportation industries were significantly higher than 20 industries.

Since the 1950s, the significance model is a widespread technique in experimental studies of global trade patterns. It was impressed by the general significance regulation, according to the magnetism among global bodies is assessed by its weight and the space hows among them. In the international political economy framework, the model stresses that trade volumes enhance the financial mass and nearness between economic partners anywhere dimension is calculated as value and proximity by geological space (Chen et al., 2011). This research has exposed that the gap is essential. Almost world trade. A quarter of the countries are important among countries that share a land border and are less than 3,000 km away from their semi-global trade partners. This highlights the Silk Road's original nature because it is apparent to incorporate far-away trade nodes using the railway system (Nunn and Trefler, 2014; Nazarko et al., 2017).

There are various points of view for trade relaxation. Free of charge trade helps to avoid losses in management potency related to economic policy (Balassa, 1985). On the other hand, in the Silk Road case, a different interpret the problems of transport infrastructure and trade liberalization is required (Shaikh et al., 2016; Nunn and Trefler, 2014). As a result of the Silk Road, trade integration can cause advantageous effects for the collaborating country and, therefore, discrimination against those who exclude themselves from the project (Marinov, 2015; Shaikh et al., 2016). The discriminatory effects are significantly felt by the country, for those trade associations with the European countries, China is an alternative contributor to the Silk Road. The researched countries were established each in the cluster of the beneficiary, which can see the current of savings and therefore enhanced their aggressiveness etc. because of the Silk Road, and the assembly of the countries, whose economies can suffer from this project (Tan and Hui, 2011; Shaikh et al., 2016; Wang, 2015).

### 3.2 Public opinion about the silk road

The present analysis can be secondary. Although public estimation is dependable with the majority of points of reference that have worked well; the authorized media have liberated the online and private estimation. It can be educated from the additional survey that the Silk Road policy has not been popularized in the community. In the parent study base on a trial survey of Pakistani scholars reading in China, 79% of the respondents were male and 21% of respondents were women students presented in Table 4. The public contribution is not sufficient; After some major initiatives by Pakistan, the methods of public participation are only nationally complex and argumentative. The conclusion that has the same view as David et al. (2016) and Shaikh et al. (2016), which is the community has no fixed judgment, regarding the complex and inaccessible foreign rule, immediately has mood swing and present an approach of indifference. Community estimation lacks the support of information and value arrangement, presenting a self-contradictory quality. It is hard to agree with a country's foreign rule and is simply affected by the estimation leader's political tendency. And Table 4 is related to the source of information about the China-Pakistan Karakoram Highway (KKH) 50% of respondents heard, regarding it from social media outlets (Zhang, 2014). The other students' sources of information were community news (28%), global conferences (9%), university courses (4%), and government promotions (8%).

**Table 4:** Respondents’ gender.

Options	Male	Female	Level of study		
			Undergraduates	Masters/M. Phil	Ph.D
Total Number of peoples	300	300	300	300	300
Ratio (%)	79%	21%	85%	0.94%	95%

**Figure 4:**
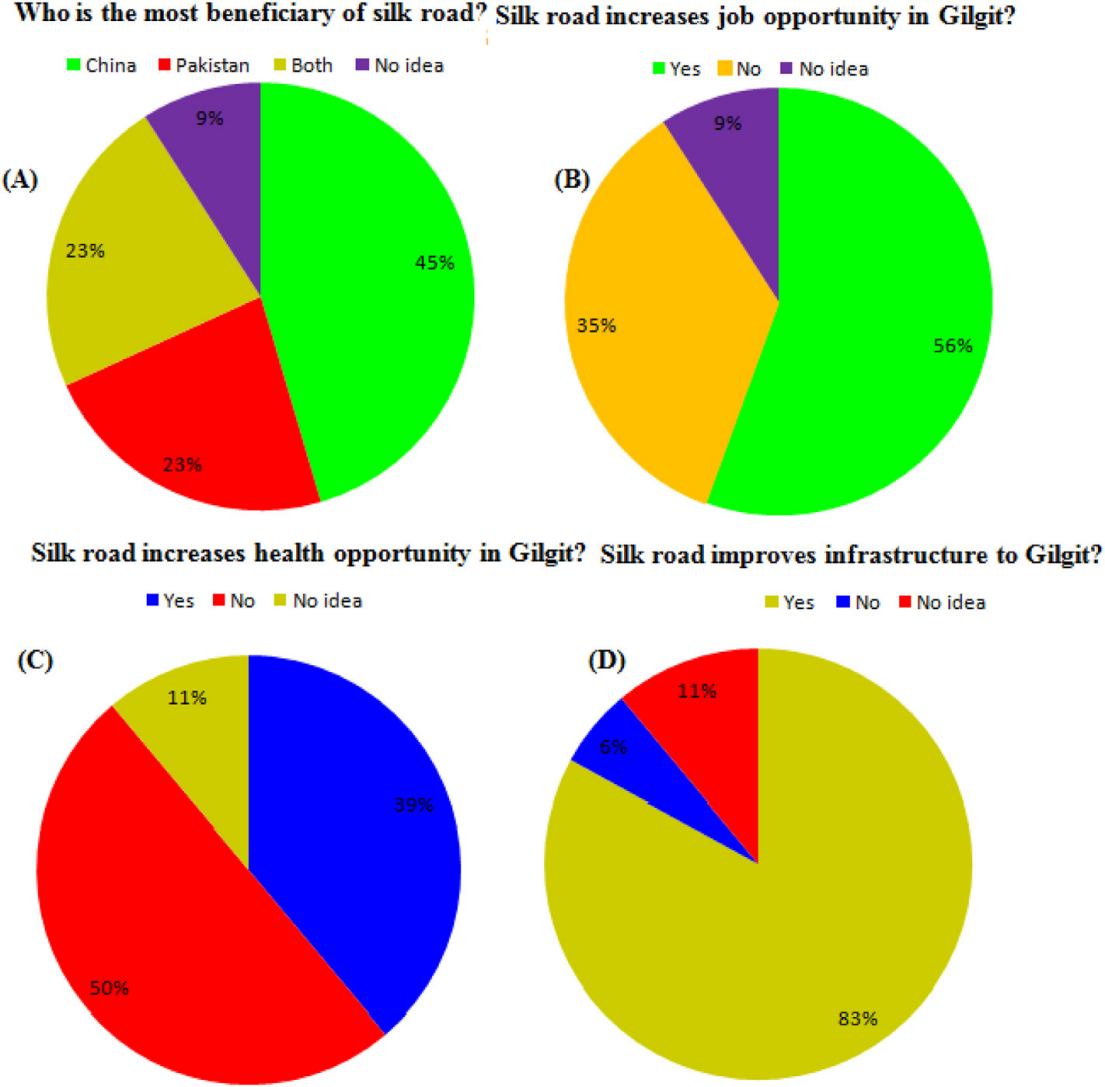
(ABCD) shown the Silk Road development performance in Gilgit, Pakistan.

Public estimation is shown in (Figure 4ABCD) that respondents were asked the problem “whom do you think will advantage most from the materialization of the China-Pakistan Karakoram Highway (KKH) Silk Road?”, 45% of respondents believe that most of the benefits of the operationalization of the China-Pakistan Karakoram Highway (KKH) Silk Road scheme will be taken by China. In contrast, 23% of the remaining respondents believe that Pakistan will benefit the most, 23% reaming respondents believe that in both countries, 9% of the respondents shape they didn't know who will benefit most from the plan is accessible in (Figure 4A) (Appendix A, Appendix B, and Appendix C). Does Silk Road increase job opportunities in Gilgit? 56% of the public estimated the silk road enhanced, 35% No and 9% have no idea about the increasing the job opportunity in Gilgit (Figure 4B). Does Silk Road increase health opportunities in Gilgit? The 39% public estimation silk road enhanced, 50% No and 11% have no idea about the increasing the health opportunity in Gilgit (Figure 4C). Does Silk Road improve the infrastructure of Gilgit? The 83% public estimation Silk Road enhanced 6% No and 11% have no idea about improving Gilgit's infrastructure (Figure 4D).

**Figure 5:**
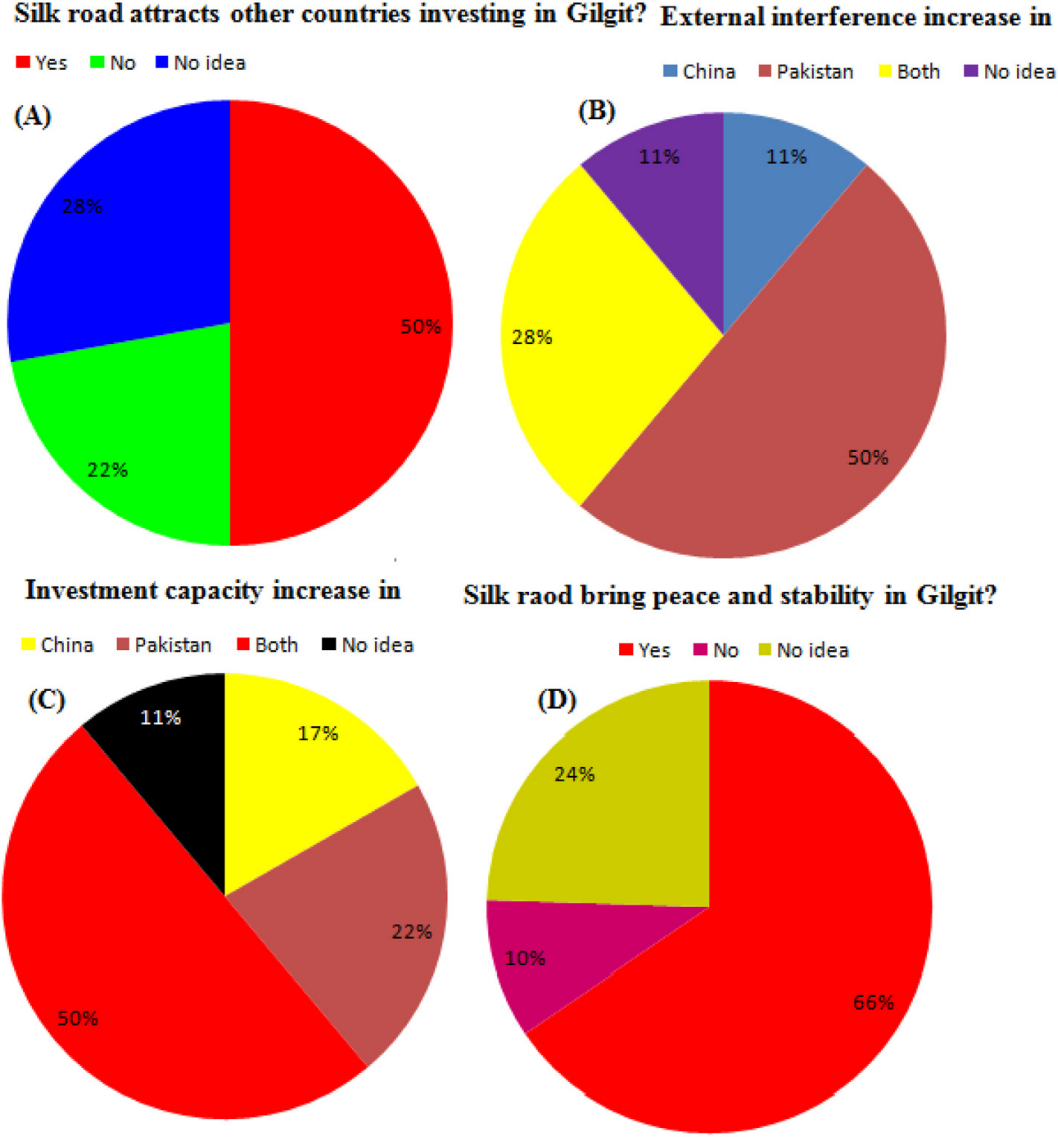
(ABCD) shown the Silk Road development performance between the two countries.

Does Silk Road attract other countries to invest in Gilgit? 50% public estimation Silk Road attracts other countries, 22% No and 28% have no idea about other countries investing in Gilgit (Figure 5A). Silk Road external infrastructure increased in which country? The 50% public estimation of Silk Road external infrastructure increased in Pakistan, 11% in China, 28% in Both countries, and 11% have no idea about external infrastructure (Figure 5B). Silk Road investment capacity increased in which country? The 22% public estimation of Silk Road investment capacity increased in Pakistan, 17% in China, 50% in Both countries, and 11% have no idea about investment capacity increased (Figure 5C). Does Silk Road bring peace and stability to Gilgit? 66% public estimated yes, 10% no, and 50% have no idea about Silk Road brings peace and stability to Gilgit (Figure 5D) (Appendix A, Appendix B, and Appendix C).

**Figure 6:**
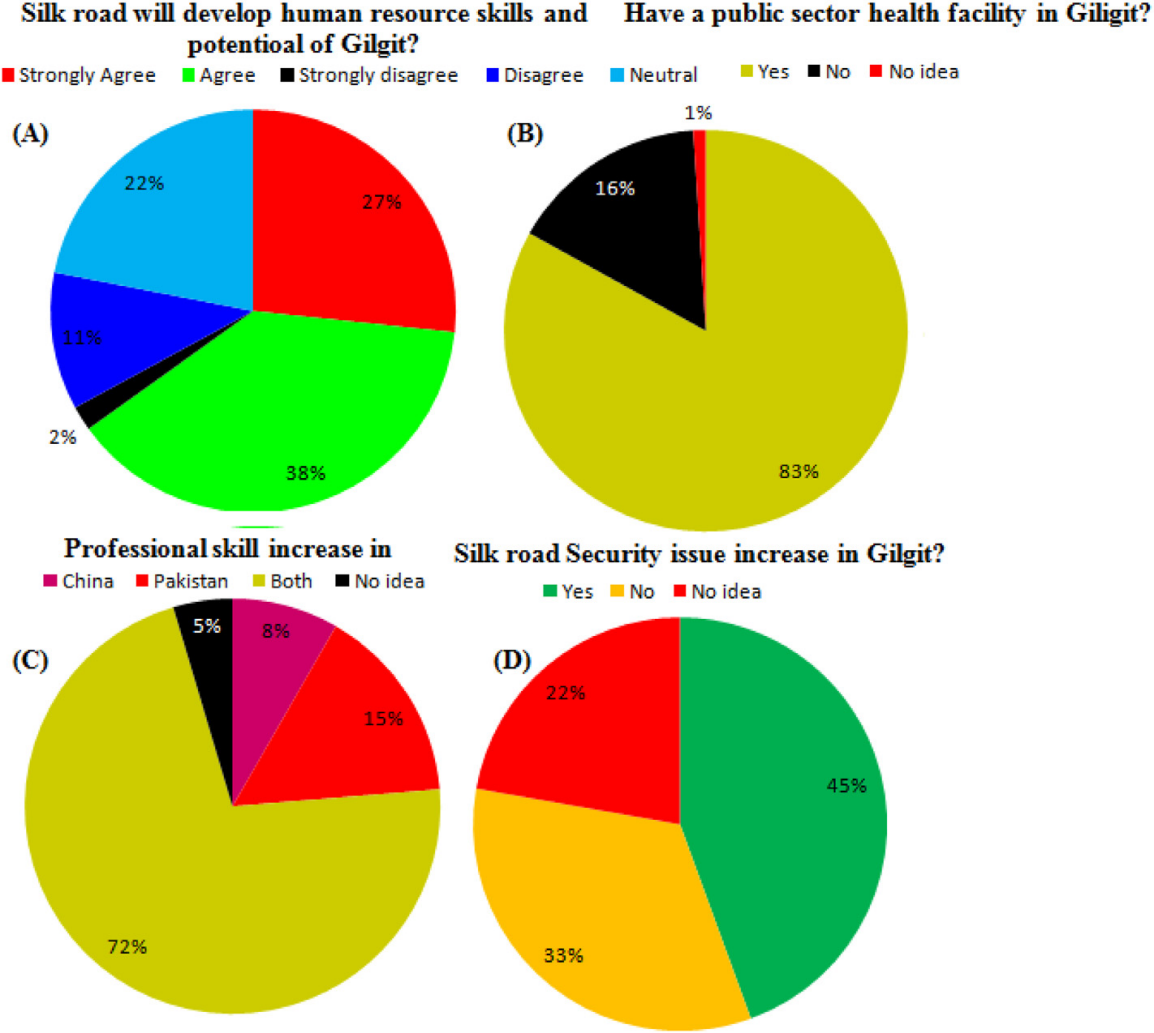
(ABCD) shown the Silk Road development performance in public sector of Gilgit Pakistan.

Will silk road develop human resource skills and positional of Gilgit? 27% public estimation has strongly agreed, 38% have agreed, 2% strongly disagree, 11% disagree, and 22% have a neutral opinion (Figure 6A) (Appendix A, Appendix B, and Appendix C). Have a public sector health facility in Gilgit? 83% of public opinion yes, 16% have no, and 1% have no idea (Figure 6B). Where do professional skills increase? 8% of students opinion professional skills increase in china, 15% in Pakistan, 72% in both countries, and 5% have no idea (Figure 6C). Silk Road security issue increase in Gilgit? 45% of students opinion yes, 33% have no, and 22% have no idea (Fig: 6D).

Given the above data investigation, the public judgment is reliable with conventional direction and the administration occupation of public judgment direction has worked better. The representative media has conquered online judgment, and individual judgment is tough to discover (Khan et al., 2013). The public concentrates on two proceedings and gives positive comments; 1st is Pakistan intends to set up 10,000 armed protection forces for the CPEC. The 2nd is Pakistan administration legitimately transferred the correct to use the Gwadar port without charge trade zone to China (Li, and Hans-Jörg, 2017). The natural history of the CPEC approach is a supporting and ambassadorial affair with no disagreement caused by a nationalized complex; therefore, a lot of people do not know or anxiety about this approach. Consequently, Chinese people will include a confident sense of national pride to enhance economic corridor manufacturing development awareness. Deling et al. (2016) reported that the University student's opinion of the CPEC could be educated through a survey questionnaire. Most of them consider that CPEC might get better the financial expansion in both counties, defend the Chinese energy reserve and produce a high-quality situation for the surrounding situation, on the other hand, there are immobile several community doubts about the vast investment that could become taxpayers frightening and there is no clear economic advantage. Besides, safety is also extremely worthy of anxiety anxiety (Wang, 2015; Zhang, 2014).

### 3.3 Significance and challenges of China-Pakistan silk road (CPSR)

Following the demographic questions, the respondents were asked about the importance of social media's challenges and significance (Appendix C).

Regarding the importance of the Silk Road, most of the respondents believed that the planned projects were a practical step in creating employment opportunities, providing infrastructure, and encouraging the flow of domestic and international investment in Pakistan. It will have a significant effect. Although energy security and peace stability drew little attention from respondents, respondents were reluctant to influence the scheme to save the international financial and political situation of China and Pakistan. Regarding the significance of the recognized challenges in influencing the project process, the root of the passageway is measured to be the most significant reason for the disruption of projects. Bureaucratic and corrupt municipal organizations and Pakistan's security issues were also factors that the Pakistani government needs to consider.

Alternatively, It may be a condition that public estimates will hardly be considered when the government formulates a chief foreign policy. The government also seldom gives the community the confidence to participate, which makes the public estimate a matter of the Daewoo document. It is also China's basic one-year policy-making system based on a high-level proposal. The administration will make additional adjustments to the following success development. It is difficult to decide whether this system is correct or not. However, as citizenship awareness is regularly better at present, this study recommended that government generally ask for public estimation before the policy is introduced. The administration also required to construct a relationship between the government and the community people. The government also needed to build a relationship between the government and the public.

## 4 Limitations

The study's limitations are significant mentioning, such as the small population used in the questionnaire survey, which might have affected the findings' reliability. Further studies are still needed associated matters. In addition to strengthening the time-tested economic partnership between the two countries, the CPSR is expected to transform China's economic and political situation in the world.

## 5 Conclusions

This paper analyzes China-Pakistan Karakoram Highway (KKH) Silk Road's challenges by considering the sample survey's public sentiment. The public sentiment results that say both countries are equal benefits because China have increased sale or marketing and in Pakistan, the China-Pakistani economic corridor project bring the international trade, healthcare, self-efficacy, and social performance facility to Gilgit city, Pakistan The results of the analysis show a better knowledge of the communication between the project and social media, and how the administration should include community estimates in its completion. Most importantly, although some matters are measured as a threat, the Pakistani people are optimistic about the China-Pakistan Karakoram Highway (KKH) Silk Road. Based on the fundamental challenges, this article presents a 'vision' that the Government of Pakistan must ensure its economic and political goals in negotiations within the CPSR. The people of Pakistan have implications to avoid this misunderstanding which is an obstacle to the goals of both the countries. In addition, the transit oil and gas pipeline with 4,000 km of coverage will ensure a sustainable energy supply for the demanding Chinese economy. This Karakoram Highway will make sure in the economic recovery of the eastern and southern parts of the Asian region and the backward areas in China and Pakistan. In addition to strengthening the time-tested economic partnership between the two countries, the China-Pakistan Silk Road is expected to transform China's economic and political situation in the world.

### 5.1 Practical implications

The majority of respondents in the survey had a positive approach to the CPSR. The research findings are beneficial for (CPSR) stakeholders and research scholars doing projects on the impact, growth, and prospects of (CPSR). This study will help China intensify its products to the Middle East, Africa, and Europe. The (CPSR) is believed to create a shift in China's economic and political position in the world.

## 6 Recommendation and Limitations

The study's limitations are significant mentioning, such as the small population used in the questionnaire survey, which might have affected the findings' reliability. Further studies are still needed associated matters.

1. In future the China-Pakistan Silk Road connected the Africa, Europe, and the Middle East countries.
2. Economic Transformation Initiative Gilgit-Baltistan programmers design report between a Rock and a Hard Place.
3. Development and challenges in the hospitality and tourism sector
4. Livestock management based trainings in the study area can elevate the capacity building of livestock farmers.

## Declarations

### Author contribution statement

Shakela Naz: Conceived and designed the experiments; Performed the experiments; Analyzed and interpreted the data; Wrote the paper.

Wu Yeyan: Performed the experiments; Analyzed and interpreted the data.

Liu Zhe: Contributed reagents, Materials, Analysis tools or data; Wrote the paper.

Keyao Ren: Contributed reagents, materials, Analysis tools or data; Wrote the paper.

Prof. Yu Wenjie Conceived and designed the experiments; Wrote the paper.

### Funding statement

This research did not receive any specific grant from funding agencies in the public, commercial, or not-for-profit sectors.

### Data availability statement

Data included in article/supp. material/referenced in article.

### Declaration of interest’s statement

The authors declare no conflict of interest.

### Additional information

No additional information is available for this paper.

## References

[bib1] Alhassan G.N., Adedoyin F.F., Bekun F.V., Agabo T.J. (2021). Does life expectancy, death rate and public health expenditure matter in sustaining economic growth under COVID-19: empirical evidence from Nigeria?. J. Publ. Aff..

[bib2] Balassa B. (1985). Exports, policy choices, and economic growth in developing countries after the 1973 oil shock. J. Dev. Econ..

[bib3] Chen Z., Ge Y., Lai H. (2011). Foreign direct investment and wage inequality: evidence from China. World Dev..

[bib4] David H., Dorn D., Hanson G.H. (2016). The China syndrome: local labor market effects of import competition in the United States. American Economiv Reviwe.

[bib5] Deling H., Diren L., Tiantian H. (2016). Analysis of public opinion about China- Pakistan economic corridor. J. Appl. Sci..

[bib6] Ejdys J. (2017). New Silk Road–a weak or a strong signal?. Proc. Eng..

[bib8] Hossain E., Rana J., Islam S., Khan A., Chakrobortty S., Ema N.S., Bekun F.V. (2021).

[bib9] Khalil J., Perveen S. (2015). Gwadar-Kashgar economic corridor: challenges and imperative for Pakistan and China. J. Polit. Studies.

[bib10] Khan A.D., Ahmed M., Malik O.M. (2013). Pak-China economic alliance to bring prosperity in region. Internat Rev. Manag. Bus. Res..

[bib11] Li Y., Hans-Jörg S. (2017). Trade and the new silk road: opportunities, challenges, and solutions. J. Chin. Econ. Bus. Stud..

[bib12] Marinov E. (2015). Economic determinants of regional integration in developing counties. Int. J. Bus. Managment.

[bib13] Masood S. (2008).

[bib14] Nazarko J., Czerewacz-Filipowicz K., Kuźmicz K.A. (2017). Comparative analysis of the Eastern European countries as participants of the new silk road. J. Bus. Econ. Manag..

[bib15] Nunn N., Trefler D. (2014). Handbook of Internation Economics.

[bib16] Oziewicz E. (2015). Gdansk Studies of East Asia.

[bib17] Shaikh F., Ji Q., Fan Y. (2016). Prospects of Pakistan-China energy and economic corridor. Renew. Sust. Energy Rev..

[bib18] Tan X., Hui C. (2011). Public opinion and foreign policy realism and liberalism genre comparison. J. Int. Studies.

[bib19] Wang P. (2015). China-Pakistan economic corridor: the flagship of one zone and one road. Foreign Invest. China.

[bib20] Zhang C. (2014). Building of China-Pakistan economic corridor: opportunities and challenges. South Asian Stud. Quart..

