# Supplementary material for: China-Pakistani economic corridor project bring the international trade, healthcare, self-efficacy, and social performance facility to Gilgit city, Pakistan
Source: Heliyon. 2022 Sep 3;8(9):e10523. doi: 10.1016/j.heliyon.2022.e10523 (PMC9468398; doi:10.1016/j.heliyon.2022.e10523)
Supplement: Supplementary data [file mmc1.doc]

## Supplementary data

**China-Pakistani Economic Corridor Project Bring the International trade, Healthcare, Self-efficacy, and Social Performance Facility to Gilgit City, Pakistan**

I am a Ph. D Scholar at Department of History, Nanjing University, China. This research project guarantees respondent confidentiality. All data will be used in a form that will make it impossible to determine the identity of the individual responses. That is, the survey responses will not be integrated, analyzed, or reported in any way in which the confidentiality of the survey responses is not absolutely guaranteed.

**Questionnaire (Appendix A)**

1. Gender: Male Female
2. Qualification: BA/BSC MA/MSC M.Phil./MS Ph.D.
3. Age: 1. 25-35 2. 36-45 3. 46-55 4. 56-70

| S.NO | Research Question | |  | |  | |  | | |  | | |  | |  | |
| --- | --- | --- | --- | --- | --- | --- | --- | --- | --- | --- | --- | --- | --- | --- | --- | --- |
| **Gilgit cultural History** | | | | | | | | | | | | | | | | |
| 1. 1 | | How many Sects in gilgit? | | 1 | | 2 | | | 3 | | 4 | | | 5 | |  |
| 1. 2 | | Which sect do you follow? | | Sunni | | Shia | | | Ismaili | | Nurbukhshi | | | Others | |  |
| 1. 3 | | Do you think that choice of dress is any way linked to religion? | | Yes | | No | | |  | |  | | |  | |  |
|  | | What is the source of basic religious education in your area? | | Parents | | School | | | Maddrasa | | Immambargah | | | Jumat  Khana | | Private institution |
|  | | What is the common difference among Deobandi, Barelvi, Shia and Ahl-e-Hadees sects? | | Interpretation | | Fundamental  Religious differences | | | Political  differences | |  | | |  | |  |
|  | | Do you listen to music? | | Yes | | No | | |  | |  | | |  | |  |
|  | |  | |  | |  | | |  | |  | | |  | |  |
| 1. 3 | | The most famous and traditional food of Gilgit? | | Chapshuro | | Mamtu | | | Sharadi | | Harissa | | | Sharbat | | Chupati |
| 1. 3 | | Gilgit is a safe place for foreigners? | | Yes | | No | | | No Idea | |  | | |  | |  |
| 1. 4 | | Majority of public in Gilgit are educated, do you agree? | | Strongly Agree | | Agree | | | Neutral | | Disagree | | | Strongly Disagree | |  |
| 1. 5 | | What kind of dialects are speaking in Gilgit? | | Balti Dialect | | Gujari Dialect | | | Changthang Dialect | | Khowar Dialect | | | Ladakhi Dialect | | Purgi Dialect |
| 1. 6 | | The famous sports of Gilgit? | | Polo | | Football | | | Volleyball | | Hockey | | | Folk dances | | Cricket |
| 1. 7 | | What type of agricultural product bumper harvest in Gilgit? | | Fruit | | Wheat | | | Maize | | Rice | | | Barley | | Beans |
| 1. 8 | | The Most domesticated animals / Livestock in Gilgit? | | Sheep/Goat | | Cows/Buffalo | | | Hores/Donkey | | Yak | | | Poultry | | Other |
| 1. 9 | | How the weather of Gilgit? | | Polluted | | Un polluted | | | Both | | No Idea | | |  | |  |
| 1. 10 | | The traditional work in arts and crafts of Gilgit. | | Stone work | | Embroidered wallets work, | | | key chains work | | Caps making work | | | other product work | |  |
| 1. 11 | |  | | | | | | | | | | | | | | |
| 1. 12 | | Do you agree with the statement that the number of Tourists in Gilgit is increasing? | | yes | | no | | No idea | | | |  | |  | |  |
| 1. 13 | | Highest ratio of tourists in Gilgit is? | | Foreigners | | Pakistani | | Both | | | |  | |  | |  |
| 1. 14 | | The Highest Peaks of Gilgit | | K2 | | Nanga Parbat | | Tarichmare | | | | No idea | |  | |  |
| 1. 15 | | What are the famous places of Gilgit? | | Hushe Valley | | Bagrote Valley | | Phandar Valley | | | | Gupis Valley | | Satpara Lake | | Fairy Meadows |
| 1. 16 | | Famous household edibles are? | | Home-grown vegetables | | Vegetables bought from market | | Pulses | | | | Others (wheat, rice, dairy, etc.) | |  | |  |
| 1. 17 | | What is your average Income in percentage? | | 20-30% | | 30-40% | | 40-50% | | | | 50-60% | | 60-70% | | 70-80% |
|  | | Political affiliation of majority of people in Gilgit is? | | PPP | | PLM | | PTI | | | | mixed | |  | |  |
| 1. 19 | | In which weather people like to visit giligit? | | Spring | | Summer | | Autumn | | | | Winter | |  | |  |

**Questionnaire (Appendix B)**

|  | **The beneficiary of Silk Road?** | | | | | | |
| --- | --- | --- | --- | --- | --- | --- | --- |
| 1. 9 | Who is the most beneficiary of Silk road? | China | Pakistan | Both | No Idea |  |  |
| 1. 10 | Silk road increases Job opportunity in Gilgit? | yes | no |  |  |  |  |
| 1. 11 | Silk road increases health opportunity in Gilgit? | Yes | No | No Idea |  |  |  |
| 1. 12 | Silk road improves Infrastructure to Gilgit? | Yes | No | No Idea |  |  |  |
| 1. 13 | Silk road brings peace and stability in Gilgit? | Yes | No | No Idea |  |  |  |
| 1. 14 | Silk road attracts other countries investing in Gilgit? | Yes | No | No Idea |  |  |  |
| 1. 15 | Silk road will develop human resource skills and potential of Gilgit | Strongly agree | Agree | Disagree | Neutral |  |  |
| 1. 18 | Have a public sector health facility in Gilgit? | Yes | No |  |  |  |  |

**Questionnaire (Appendix C)**

|  | **Challenging factors to Gilgit** | | | | | | |
| --- | --- | --- | --- | --- | --- | --- | --- |
| 1. 19 | Security issue increase in Gilgit? | Yes | No | No Idea |  |  |  |
| 1. 20 | Professional skill increase in | China | Pakistan | Both | No Idea |  |  |
| 1. 21 | Investment capacity increase in | China | Pakistan | Both | No Idea |  |  |
| 1. 22 | System of administration increase in | China | Pakistan | Both | No Idea |  |  |
| 1. 23 | External interference increase in | China | Pakistan | Both | No Idea |  |  |
